# Adenosine-Induced NLRP11 in B Lymphoblasts Suppresses Human CD4^+^ T Helper Cell Responses

**DOI:** 10.1155/2020/1421795

**Published:** 2020-08-03

**Authors:** Irem Ozel, Ilgin Akkaya, Ece Oylumlu, Goksu Uzel, Ceren Ciraci

**Affiliations:** College of Science and Letters, Molecular Biology and Genetics Department, Istanbul Technical University, Istanbul 34469, Turkey

## Abstract

NLRP11 is a member of the PYD domain-containing, nucleotide-binding oligomerization-domain (NOD-) like receptor (NLR) family. The true stimulus of NLRP11 is still unclear to date, so the current study is built upon NLRP11 induction via adenosine stimulation and that activation can shape adaptive immune responses in a caspase-1-independent manner. We examined the regulation and mechanism of adenosine responsiveness via NLRP11 in human Daudi Burkitt's B lymphoma cells and their effects on human peripheral CD4^+^ T lymphocytes from healthy individuals. NLRP11 was significantly upregulated after induction with adenosine at both the mRNA and protein levels, which led to the interaction of endogenous NLRP11 with the ASC adaptor protein; however, this interaction did not result in the activation of the caspase-1 enzyme. Furthermore, cocultures of NLRP11-expressing Burkitt's lymphoma cells and naïve human peripheral CD4^+^ T lymphocytes had reduced IFN-*γ* and IL-17A production, whereas IL-13 and IL-10 cytokines did not change. Interestingly, IFN-*γ* and IL-17A were recovered after transfection of Burkitt's lymphoma cells with siRNAs targeting NLRP11. Concomitant with NLRP11 upregulation, we also exhibited that adenosine A_2B_ receptor signaling induced two phosphorylated downstream effectors, pErk1/2 and pAkt (Ser473), but not pAkt (Thr308). Taken together, our data indicate that adenosine is a negative regulator of Th1 and Th17 responses via NLRP11 in an inflammasome-independent manner.

## 1. Introduction

The NOD-like receptor (NLR) family of Pattern Recognition Receptors (PRRs) is composed of cytosolic proteins that sense intracellular PAMPs and DAMPs and initiate an innate immune response leading to inflammation and/or cell death. The NLR family is comprised of more than 20 intracellular immune receptors that share structural domains with different functional specializations. The NLR proteins have an N-terminal pyrin domain (PYD) or caspase recruitment domain (CARD) that can interact with other proteins, a central nucleotide-binding oligomerization (NOD) domain for self-oligomerization, and a C-terminal leucine-rich repeat (LRR) domain that recognizes cytosolic PAMPs and DAMPs [1].

NLRs are grouped into four structurally similar subfamilies, namely, NLRA, acidic domain containing; NLRB, baculoviral inhibitory repeat (BIR) domain containing; NLRC, caspase activation and recruitment domain (CARD) containing; and NLRP, pyrin domain (PYD) containing, as well as, NLRX, which has no significant homology to the N-terminal domain of any other member of the NLR subfamily [2]. Although NLRP11 is commonly considered a primate-specific NLR [3], rabbits, placentals, bats, pigs, and lemurs also express different isoforms of NLRP11 with amino acid sequence identities ranging from 45.6% to 58.6%. Additionally, sequence comparison analysis revealed that human NLRP11 has the closest amino acid sequence identity to NLRP4 of the nonprimate species *Tupaia chinensis* (36%) and *Mus musculus* (33.5%).

The pyrin-containing NOD-Like Receptor (NLRP) subfamily of NLR proteins is well known for its ability to form multiprotein complexes called inflammasomes through interactions with the ASC adaptor protein and pro-caspase-1 enzyme [4]. Caspases are a family of cytosolic cysteine proteases that regulates diverse cellular mechanisms such as inflammation and apoptosis. Thus, their activation is tightly controlled by various intrinsic and extrinsic signals. Caspase-1 is present in the cytosol of phagocytic cells as an inactive zymogen called pro-caspase-1 [5]. The activation of pro-caspase-1 is concurrent with the assembly of an inflammasome complex in the cytosol. Once localized in the inflammasome complex, pro-caspase-1 cleaves itself into an enzymatically active form. The active caspase-1 then cleaves the proinflammatory cytokines IL-1*β* and IL-18 into their mature and secreted forms. After secretion, these proinflammatory cytokines participate in various immune responses such as inflammation and regulation of the adaptive immune response [6]. Apart from the initiation of inflammatory responses, the NLR proteins regulate some aspects of adaptive immune responses such as cytokine production by lymphocytes and T cell proliferation and differentiation via inflammasome complexes [6].

Activation of the inflammasome complex in response to sterile insults is another facet of the immune system, which leads to maintenance of homeostasis and regulation of tissue repair. DAMPs are endogenous molecules that are released after cellular stress, tissue damage, ischemia, hypoxia, and inflammation [7]. These molecules, which include purine metabolites (extracellular ATP, adenosine, and uric acid), high mobility box 1 (HMGB1), heat shock proteins (HSPs), and Reactive Oxygen Species (ROS), can also be recognized by NLRs in the promotion of inflammasome complex formation and subsequent inflammation [8]. Adenosine is an endogenous purine nucleoside, which has crucial regulatory effects on the immune system [9]. The extracellular concentrations of adenosine can increase under several conditions including inflammation, ischemia, and hypoxia [10]. Adenosine exerts its effect through four types of adenosine receptors, namely, the adenosine A_1_, A_2A_, A_2B_, and A_3_ receptors [11]. The impact of adenosine on immune responses is bidirectional and its effects on immune cells vary depending on its concentration which activates the adenosine receptor [12]. Adenosine binding to A_2A_ receptors blocks the release of proinflammatory cytokines such as IFN-*γ* in CD4^+^ murine T cells, and it induces the production of anti-inflammatory cytokines such as IL-10 in macrophages [13, 14]. Of particular interest, A_2B_ receptors have been shown to be involved in numerous inflammatory diseases such as colitis, ischemia-driven inflammation, COPD, acute lung injury, and vascular disease [15] .

Because of their central role in the inflammatory responses, NLR proteins are associated with several inflammatory and autoinflammatory diseases, and therefore are targeted for the treatment of these diseases [16]. For example, several negative regulators that inhibit the formation of NLRP3 inflammasome complexes and suppress inflammatory responses have been documented [17]. NLRP11, a member of this family, is highly expressed in the testes, ovaries, and lungs and in various other human tissues. In the immune cell context, NLRP11 mRNA is abundant in monocytes and B-lymphocytes. Interestingly, the mRNA level of NLRP11 is remarkably high in Daudi cells (a human Burkitt's lymphoma cell line) and myeloid THP1 cells, whereas it is low in epithelial cell lines including HeLa [3]. Although the function of NLRP11 is largely unknown, recent studies have shown that NLRP11 acts as a negative regulator of NF-*κ*B activation by promoting the ubiquitination and degradation of the TRAF6 protein and thus ultimately inhibiting the MyD88 signaling pathway [18]. In another study, Qin et al. demonstrated that NLRP11 impedes the antiviral immune response by disrupting the MAVS signalosome, thereby suppressing the production of the type I interferon via TRAF6 degradation [19].

Since their discovery, NLRs have drawn considerable attention for their ability to form inflammasomes and also for acting independently. However, very few studies have addressed the roles of NLRP11 in shaping T cell immune responses. Basal expression of NLRP11 in B lymphoblast cell lines led us to work with primate-specific endogenous NLRP11 rather than overexpressed NLRP11, which made Daudi cells a good model for further analysis of adaptive immunity. We herein report that adenosine significantly upregulates NLRP11 expression. Although, adenosine-induced NLRP11 did not lead to the secretion of proinflammatory cytokines from Daudi cells, NLRP11 interacted with the ASC adaptor protein upon adenosine stimulation. Moreover, adenosine treatment reduced not only the caspase-1 enzyme activity but also the intracellular active caspase-1 and mature IL-1*β* protein levels. Additionally, we examined the roles of NLRP11 in orchestrating CD4^+^ T cell responses by coculturing NLRP11-expressing Daudi cells and naïve human peripheral blood CD4^+^ T lymphocytes. Our study suggested a novel role for NLRP11 in the suppression of Th1 and Th17 responses in an inflammasome-independent manner. Of the adenosine receptors we examined, the A_2B_ receptor had higher gene expressions than the A_1_, A_2A_, and A_3_ adenosine receptors in Daudi cells. Finally, we found that phosphorylation of two downstream effectors of adenosine A_2B_ receptor signaling, namely, pERK1/2 and Akt/pAkt, was concurrent with NLRP11 upregulation. Taken together, our data indicate that adenosine is a negative regulator of Th1 and 17 responses via NLRP11.

## 2. Materials and Methods

### 2.1. Cell Cultures and Stimulation of Cells with Different Agonists and Inhibitors

The Daudi cell line [20] was cultured in RPMI 1640 medium (PAN) supplemented with 10% heat-inactivated newborn calf serum, 2 mM glutamine, 1 mM sodium pyruvate, 0.1 mM nonessential amino acids, 100 U/ml penicillin, 100 *μ*g/ml streptomycin, 10 mM HEPES at 37°C, and 5% CO_2_. Cells were plated in 75 cm^2^ tissue flasks (CELLSTAR, Greiner Bio-One), and cultures were split every 3 days. Cell viability was >90% by trypan blue exclusion (Sigma-Aldrich). TLR agonists were dissolved in endotoxin-free H_2_O. Cells were cultured at an initial density of 2 × 10^6^ cells/ml into 25 cm^2^ tissue flasks and kept overnight in the incubator, then stimulated with the following: 50 ng/ml lipopolysaccharide (LPS) from *Salmonella enteritidis* (InvivoGen); 0, 25, 50, 75, and 100 *μ*M adenosine (Sigma-Aldrich); 1 *μ*g/ml CD40L (CST); 50 ng/ml PMA (Santa Cruz); and 500 ng/ml ionomycin (Santa Cruz) or 50 *μ*M caffeine. As a control, the cells were treated with endotoxin-free H_2_O. These preparations were collected at 4 and 24 hours after stimulation. Daudi cells were also stimulated with 50 *μ*M caffeine, uridine (Sigma-Aldrich), and cytidine (Sigma-Aldrich) at 4 hours after stimulation. The protein translation inhibitor cycloheximide (CHX; Biofroxx) was resuspended in deionized water. 2 × 10^6^ cells/ml were treated with CHX (10 *μ*g/ml) for 1 hour before adenosine (50 mM) treatment. Cells were lysed 4 hours after adenosine stimulation.

### 2.2. CD4^+^ T Helper Cell Culturing and Stimulation

PBMCs were isolated from human whole blood by using Histopaque (Sigma-Aldrich) density gradient centrifugation. CD4^+^ T cells were prepared by positive selection from PBMCs using CD4 Miltenyi beads per the manufacturer's instructions (Miltenyi Biotec, San Diego, CA). The 24-well plates were coated with 1 *μ*g/ml anti-CD28 (BioLegend) and 2 *μ*g/ml anti-CD3 (BioLegend) antibodies overnight. Blood-derived human primary CD4^+^ T cells were cultured in RPMI-1640 (Sigma-Aldrich) supplemented with heat-inactivated newborn calf serum, 2 mM glutamine, 1 mM sodium pyruvate, 0.1 mM nonessential amino acids, 100 U/ml penicillin, 100 *μ*g/ml streptomycin, 5 × 10^−5^ M 2-mercaptoethanol (pH 7.3), and 2 ng/ml hrIL-2 at an initial density of 2.0 × 10^6^/well into 24-well anti-CD3- and anti-CD28-precoated 24-well plates.

### 2.3. Coculturing Daudi and CD4^+^ T Helper Cells

NLRP11 siRNA-transfected or nontransfected Daudi cells and CD4^+^ T helper cells were cocultured at a ratio of 1 : 1 with an initial density of 8.0 × 10^5^ cells/well into 24-well anti-CD3 and anti-CD28 precoated 24-well plates for 3 days at 37°C and 5% CO_2_. Cell staining with anti-CD80 and anti-CD86 (BioLegend) were assessed by flow cytometry on an Accuri C6 flow cytometer (BD Biosciences) and data analyzed with FlowJo software (Tree Star Inc., Ashland, OR).

### 2.4. NLRP11 siRNA Construction and Transfection

Three siRNA targeted towards NLRP11 (Thermo Fisher Scientific) were used.

The NLRP11 siRNA antisense sequences used are as follows:

- siRNA #1: 5′-AUAUAUUGACAGAUAUCGC-3′
- siRNA #2: 5′-UUUAACUCGAAUCUUAUGU-3′
- siRNA #3: 5′-UUCGACAGCUGCAAGGUGG-3′

A universal negative control siRNA (Stealth RNAi™ siRNA Negative Control LO GC), not homologous to anything in the vertebrate transcriptome and tested to not induce stress, was used to normalize relative gene inhibition of the target gene.

Twenty-four hours before transfection, 4‐8 × 10^5^ cells in 500 *μ*l growth media without antibiotics were transferred onto 24-well plates and transfected with 100 pmol NLRP11 siRNA S1, S2, S3, a combination of S1-S3, or a nonsense negative control, using the chemical transfection reagent Lipofectamine RNAiMAX (Thermo Fisher Scientific) according to the manufacturer's instructions. Briefly, siRNAs were diluted in 250 *μ*l Opti-MEM (Gibco) and mixed gently. 1.5 *μ*l Lipofectamine RNAiMAX was diluted in 50 *μ*l Opti-MEM medium and combined with the diluted RNAi duplex, incubated for 20 min at room temperature, then added to each well containing cells to give a final RNA concentration of 100 pmol. Transfection efficiency was evaluated under a fluorescent microscope, using BLOCK-iT™ Alexa Fluor® Red Fluorescent Control (Thermo Fisher Scientific) at 18-24 hours post transfection. Twenty-four hours after transfection, siRNA-transfected and nontransfected Daudi cells were added onto 8 × 10^5^ human CD4^+^ T cell-containing wells at a final volume of 1 ml and incubated at 37°C in a CO₂ incubator for 3 days. Cocultures were stimulated with 50 *μ*M adenosine (Sigma-Aldrich). Supernatants were collected and assayed for IL-13, IFN-*γ*, IL-17A, and IL-10. Antibody pairs for the cytokine ELISAs were from BioLegend. Cells were harvested for RNA isolation.

### 2.5. Real-Time RT-PCR

Total RNA was isolated from samples (3 wells from 24-well plates, 3 replicates per each treatment) using RNAquous© (Ambion, Austin, TX) according to the manufacturer's instructions. All RNA samples were DNase treated with DNA-Free (Ambion, Austin, TX) according to the manufacturer's instructions before QPCR.

The mRNA expression levels of NLRP11 primers [21], IL-1*β* primers (F 5′-TGGCAATGAGGATGACTTGT-3′, R 5′-GGAAAGAAGGTGCTCAGGTC-3′), RORC primers (F 5′-TTTTCCGAGGATGAGATTGC-3′, R 5′-CTTTCCACATGCTGGCTACA-3′), T-bet primers [22], GATA3 primers (F 5′-GTCCTCCCTGAGCCACATCT-3′, R 5′-GTGGTCCAAAGGACAGGCTG-3′), and HPRT1 primers [11] as a housekeeping gene were determined by quantitative real-time RT-PCR using QuantiTect SYBR Green RT-PCR (Qiagen, Waltham, MA) with A_1_R primers (F 5′-TGCACTGGCCTGTTCTGTAG, R 5′-CTGCCTCTCCCACGTACAAT), A_2A_R primers (F 5′-GGAGTTTGCCCCTTCCTAAG, R 5′-CTGCTTCCTCAGAACCCAAG), A_2B_R primers (F 5′-GGGCTTCTGCACTGACTTCT, R 5′-CCGTGACCAAACTTTTATACCTG), and A_3_R primers (F 5′-TCAAAGCTTGTGTGGTCTGC, R 5′-TAATTGGGGAGCACTGGAGA). Each RT-PCR reaction was run in duplicate and consisted of either 50 ng/*μ*l total RNA, 10 ml QuantiTect SYBR Green master mix, 0.25 ml QuantiTect RT mix, forward and reverse primers, and RNAse-free water for a final volume of 20 ml. The QPCR reactions were performed with a real-time PCR system (StepOnePlus; Applied Biosystems). An initial 50°C step for 30 min was followed by 95°C for 15 min and 40 cycles (94°C for 15 s, 59°C for 30 s, and 72°C for 30 s for denaturation, annealing, and extension, respectively) for all PCR amplifications. Gene slopes were determined with 10-fold serial dilutions. A melting curve from 60 to 90°C with a reading at every 1°C was also performed for each individual RT-PCR plate. The mRNA levels for the target gene corrected to those for the housekeeping gene HPRT were calculated by subtracting their corresponding Ct before and after stimulation using the following formulas: (1) before stimulation, ΔCt_control_ = Ct_target gene control_ − Ct_HPRT control_, and (2) after stimulation, ΔCt_stimulated_ = Ct_target gene stimulated_ − Ct_HPRT stimulated_. The fold change in mRNA was determined by the following formula: fold change = 2(Ct(stimulated) − Ct(control)). Experiments were performed at least twice, and one representative experiment is depicted. Results were expressed as fold change in expression of stimulated cells relative to nonstimulated cells.

### 2.6. Caspase-1 Activity

Caspase-1 activity was measured by an *in vitro* caspase detection kit according to the manufacturer's instructions (Abcam). Briefly, Daudi cells were treated with either 50 *μ*M adenosine or PBS for 4 hours. YVAD-AFC substrate was added and the cells were incubated for 2 hours at 37°C. After washing, cells were read on a fluorescent plate reader (excitation wavelength 400 nm, emission wavelength 505 nm).

### 2.7. Coimmunoprecipitation of the Endogenous NLRP11 with ASC Adaptor Molecule and Immunoblotting

Daudi cell lysates and anti-human ASC (Enzo Life Sciences, Farmingdale, NY) antibody and isotype control (IgG2b, Sigma-Aldrich) were incubated overnight at 4°C with rotation. The Pierce™ Protein A/G Magnetic Beads (Thermo Fisher Scientific) were then added to the antigen/antibody mixture and incubated at room temperature for 1 hour with rotation. The precipitated proteins were detected by immunoblotting with anti-human NLRP11 (Abcam). Samples from the same cell lysates were analyzed separately due to background issues related to different enhanced chemiluminescence (ECL) sensitivities. Electrophoresis of Daudi cell lysates was performed using the SDS-polyacrylamide gel electrophoresis (PAGE) in an 8–12% (wt/vol) polyacrylamide gel and blotted to polyvinylidenedifluoride (PVDF) membranes (Bio-Rad). To detect caspase-1, IL-1*β*, ASC, NLRP11, and *β*-actin, rabbit polyclonal anti-human caspase-1 p20 Abs (Abcam), rabbit polyclonal anti-human IL-1*β* Abs (CST), rabbit polyclonal anti-human ASC Abs (Enzo Life Sciences, Farmingdale, NY), rabbit monoclonal anti-human NLRP11 Abs (Abcam), and rabbit monoclonal anti-human *β*-actin (CST), GAPDH (CST), Vinculin (CST) were used, respectively. The membrane was visualized after incubating with HRP-conjugated anti-rabbit IgG Abs (CST) by ECL (Roche) using the ChemiDoc XRS+ System (Bio-Rad). Band intensity was detected via chemiluminescence and quantified using Image Lab Software (Bio-Rad).

### 2.8. Statistical Analysis

Statistics were performed using an unpaired Student's two-tailed *t*-test or two-way ANOVA (^∗^*P* ≤ 0.05, ^∗∗^*P* ≤ 0.01, and ^∗∗^*P* ≤ 0.001).

## 3. Results and Discussion

Members of the NLR family, including NLRP1, NLRP3, and NLRC4, have been extensively shown to contribute to inflammatory responses by forming inflammasome complexes once activated with relevant PAMPs and DAMPs in a process that subsequently leads to the secretion of proinflammatory cytokines [23]. On the other hand, multiple studies have shown the anti-inflammatory functions of NLRP12, NLRC3, and NLRX1 as negative mediators of inflammation through TLR signaling [24–26]. Although these receptors are mostly expressed and utilized by innate immune system cells, it is now known that NLRs are expressed in cells of the adaptive immune system as well [5, 27]. Therefore, the determination of molecular mechanisms shaping adaptive immunity is of high priority for understanding the nature of inflammation and developing more effective global cancer disease control approaches. We chose Daudi cells for three main reasons: (1) they serve as a model due to their ability to generate a rapid (within hours) differential gene expression response to different stimuli, (2) they have a high expression of primate-specific NLRP11 whose roles in regulating human CD4^+^ T cell responses are largely known, and (3) they express CD80 and CD86 costimulatory molecules and therefore exhibit APC characteristics. It has been suggested that NLRP11 serves as a negative regulator of inflammatory response in the innate immune system cells [3, 18, 19].

### 3.1. Adenosine Treatment Induced NLRP11 Expression at Both mRNA and Protein Levels in Daudi Cells In Vitro

**Figure 1:**
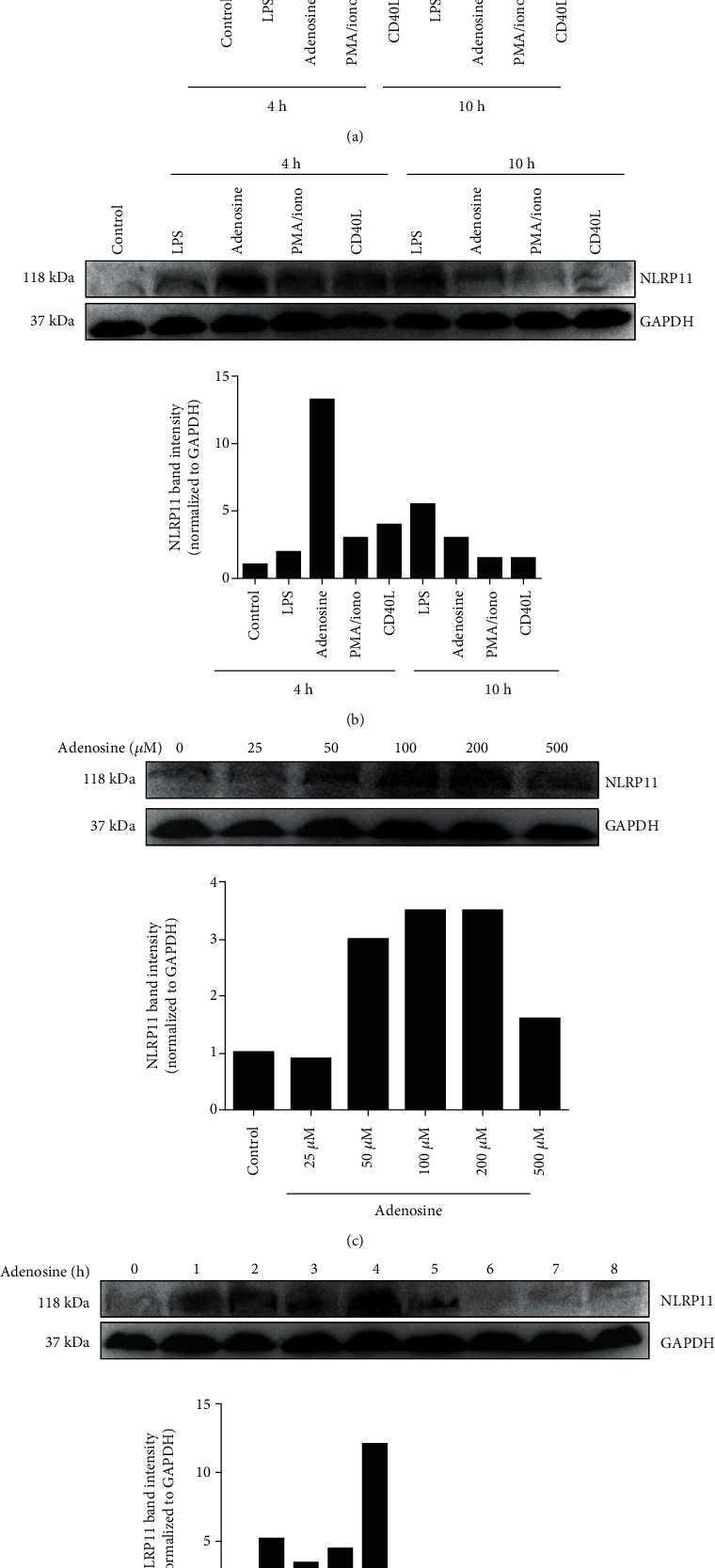
Adenosine treatment induced NLRP11 expression at both the mRNA and protein levels in B lymphoblasts. (a) NLRP11 mRNA expression after 4 and 10 hours of stimulation with PMA/ionomycin (50 ng/500 ng/ml), CD40L (1 *μ*/ml), LPS (100 ng/ml), and adenosine (50 *μ*M) (^∗^ indicates significance at *P* < 0.05; stimulated vs. nonstimulated). (b) NLRP11 protein expression after 4 and 10 hours of stimulation with PMA/ionomycin (50 ng/500 ng/ml), CD40L (1 *μ*/ml), LPS (100 ng/ml), and adenosine (50 *μ*M). (c) NLRP11 protein expression levels at 0.0, 25, 50, 100, and 200 *μ*M adenosine. (d) NLRP11 protein expression levels at 1, 2, 3, 4, 5, 6, 7, and 8 hours post stimulation with 50 *μ*M adenosine. Results are representative of four independent experiments. Student's *t*-test shows the significant difference between stimulated and nonstimulated cells. ^∗^ indicates *P* < 0.05, ^∗∗^*P* < 0.01, and ^∗∗∗^*P* < 0.001.

NLRP11 is highly expressed in Daudi cells, a human B cell lymphoma cell line, but its specific stimulant is currently unknown both *in vitro* and *in vivo* [3, 18, 19]. For this reason, we examined the responsiveness of NLRP11 to several agonists including PMA/ionomycin, CD40L, LPS, and adenosine in Daudi cells (Figure 1). To select the optimal time for harvesting cells following induction, we measured the expression level of NLRP11 mRNA after 4 and 10 hours. NLRP11 gene expression was higher in cells that were treated with 50 *μ*M adenosine than in those treated with PMA/ionomycin, CD40L, or LPS at 4 hours post stimulation (Figure 1(a)). Adenosine significantly induced NLRP11 mRNA expression when compared to nonstimulated cells (*P* = 0.02). NLRP11 mRNA expression was higher in cells at 4 hours post stimulation with adenosine than at 10 hours. We then tested the effect of adenosine stimulation at the protein level by immunoblotting (Figure 1(b)). Interestingly, Daudi cells treated with adenosine expressed higher levels of NLRP11 protein at the 4-hour time point than with any other tested stimulants observed. To determine the dose and time effect of adenosine treatment on NLRP11 protein expression, we first treated Daudi cells with 0.0, 25, 50, 100, 200, and 500 *μ*M adenosine (Figure 1(c)). Protein levels of NLRP11 were at the highest at 200 *μ*M, and then these gradually decreased with increasing doses of adenosine. We chose the minimum required concentration of adenosine (50 *μ*M) based on the cell viability (greater than 97%) for the subsequent analysis of endogenous NLRP11. Cell viability dramatically decreased (less than 60%) when the adenosine concentration was higher than 200 *μ*M. Secondly, we evaluated the importance of the duration of stimulation by harvesting Daudi cells at 1, 2, 3, 4, 5, 6, 7, and 8 hours post adenosine stimulation (Figure 1(d)). Similar to mRNA levels, adenosine treatment induced the highest levels of NLRP11 protein at 4 hours post stimulation.

### 3.2. Adenosine-Mediated NLRP11 Stimulation Involves De Novo Protein Synthesis

**Figure 2:**
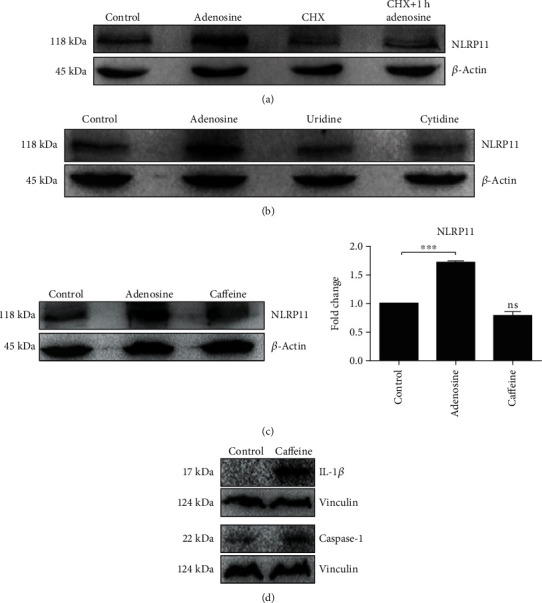
Adenosine-induced NLRP11 protein synthesis is de novo. (a) Daudi cells were left untreated or were treated with 10 *μ*g/ml CHX and then mock stimulated or stimulated with adenosine for 4 hours. (b) Cells were treated with 50 *μ*M uridine or cytidine for 4 hours. (c) NLRP11 mRNA and protein expression. (d) IL-1*β* and caspase-1 protein expressions after treatment with 50 *μ*M caffeine for 4 hours. Cell lysates were separated by SDS-PAGE and immunoblotted (IB). Results are representative of three independent experiments. Student's *t*-test shows the significant difference between stimulated and nonstimulated cells. ^∗^ indicates *P* < 0.05, ^∗∗^*P* < 0.01, and ^∗∗∗^*P* < 0.001.

Because we consistently detected that NLRP11 protein levels were upregulated by adenosine treatment, we tested the extent to which adenosine-induced NLRP11 protein was newly synthesized (Figure 2). To that end, we treated the Daudi cells with cyclohexamide (CHX), a small molecule that inhibits the elongation step of eukaryotic protein translation [28], and then treated the cells with adenosine. In contrast to nontreated cells, adenosine-induced Daudi cells in the presence of CHX had reduced NLRP11 in the lysate (Figure 2(a)), suggesting that Daudi cells quickly synthesize the NLRP11 protein following adenosine induction.

**Figure 3:**
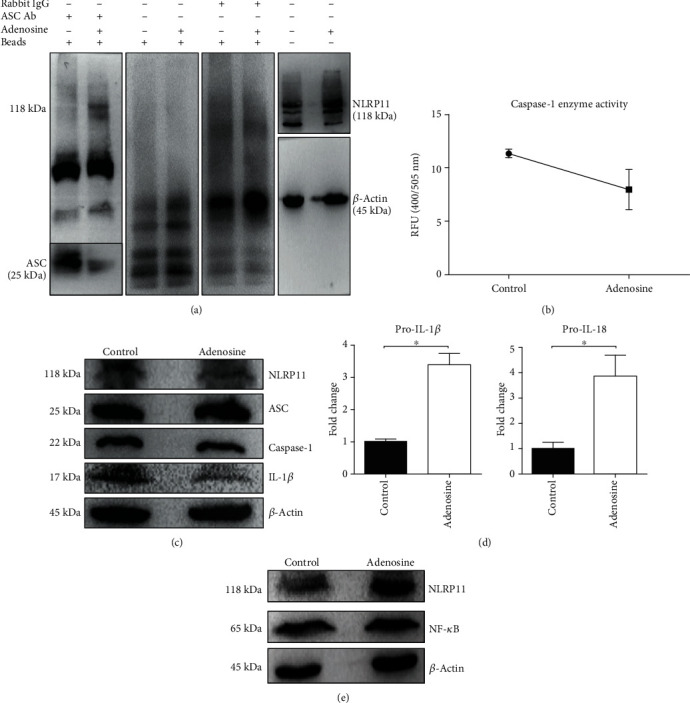
Adenosine-induced endogenous NLRP11 interacts with the ASC adaptor protein but does not lead to the activation of caspase-1 enzyme and secretion of IL-1*β*. (a) Left panel: proteins were extracted, immunoprecipitated (*IP*) via incubation with anti-ASC antibody, and followed by precipitation with protein-A/G-magnetic beads. Blots were probed with an anti-NLRP11 antibody and visualized using ECL after incubation with an anti-rat HRP-conjugated antibody. Middle panels: controls. Right lane: cell lysates were separated by SDS-PAGE and immunoblotted (IB). (b) Caspase enzyme activity: cells were treated with either 50 *μ*M adenosine or PBS for 4 hours. YVAD-AFC substrate was added and incubated for 2 hours at 37°C. Fluorescent intensity was measured on a fluorescent plate reader (excitation wavelength: 400 nm; emission wavelength: 505 nm). (c) Adenosine-treated cell lysates were separated by SDS-PAGE, and intracellular IL-1*β*, ASC, and caspase-1 levels were measured by immunoblotting. (d) Differential mRNA expressions of pro-IL-1*β* and pro-IL-18 were measured by QPCR, and data was normalized to HPRT and depicted as fold changes in the *Y* axis. Student's *t*-test shows the significant difference between stimulated and nonstimulated cells. ^∗^ indicates *P* < 0.05, ^∗∗^*P* < 0.01, and ^∗∗∗^*P* < 0.001.

Adenosine is a purine nucleoside that is largely known for its anti-inflammatory effects in the immune system, a function which might be attributable to the downregulation of IL-1*β* and caspase-1 protein after adenosine treatment. To address whether the increase in protein levels of NLRP11 is specific to adenosine, Daudi cells were stimulated with other nucleosides such as uridine and cytidine, in addition to adenosine, for 4 hours. The data from this experiment indicated that adenosine specifically induced the NLRP11 protein, while uridine and cytidine had no effect (Figure 2(b)). To further verify the effect of adenosine on NLRP11 mRNA and protein expression, cells were treated with caffeine, an antagonist of adenosine receptors. While caffeine treatment potently decreased NLRP11 protein levels (Figure 2(c)), in contrast to adenosine (Figure 3), it increased intracellular IL-1*β* and caspase-1 proteins in Daudi cells (Figure 2(d)). Collectively, these data suggest that adenosine can promote NLRP11 expression and this can be reversed by caffeine treatment.

### 3.3. Adenosine-Induced NLRP11 Interacts with ASC Adaptor Protein but Does Not Lead to the Activation of Caspase-1 Enzyme and Secretion of IL-1*β*

Research over the past few decades suggests that upon activation with specific ligands in the cytoplasm, some of the NLR members interact with the ASC adaptor protein to form multimeric protein complexes called “inflammasomes” that, in turn, mediate the recruitment of the pro-caspase-1 enzyme [29]. In the canonical pathway, the activity of caspase-1 (and caspase-11 in mice) leads to the generation of Interleukin- (IL-) 1*β* via cleavage of its proform [4]. Earlier characterization studies on NLRP11 reported that NLRP11 does not colocalize with ASC in 293T cells [30]. Furthermore, NLRP11 does not associate with ASC in transiently transfected living HeLa cells [3]. Hence, we assessed the impact of adenosine activation on NLRP11 and ASC protein interaction by coimmunoprecipitation (Figure 3(a)). Upon induction with adenosine, the endogenous NLRP11 protein precipitated with the ASC adaptor protein but did not interact with ASC in the untreated cells. To determine if this interaction resulted in inflammasome formation, as indicated by IL-1*β* secretion after caspase-1 cleavage, we measured caspase-1 enzyme activity (Figure 3(b)) and determined extracellular IL-1*β*, IL-18, and IL-6 by ELISA. Interestingly, we observed a decline in the caspase-1 enzyme activity in adenosine-treated cells as compared to nontreated cells. Consistent with the caspase-1 decrease, we did not detect any IL-1*β*, IL-18, and IL-6 secretion (data not shown). We then measured the intracellular mature caspase-1 and IL-1*β* levels after adenosine treatment by immunoblotting (Figure 3(c)). Here, we found that adenosine-induced Daudi cells had less intracellular IL-1*β* and caspase-1 protein than untreated cells. Despite the basal expression of NLRP11 and IL-1*β* in Daudi cells [31, 32] (Figure 3(c)), we clearly showed the significant difference the adenosine treatment caused throughout the experiments. Although induction with adenosine did not cause any mature IL-1*β*, IL-18, or IL-6 (an inflammasome-independent proinflammatory cytokine) secretion, we observed a differential expression of pro-IL-1*β* and pro-IL-18 mRNAs, data which is in line with the current literature [31]. The mRNA levels of pro-IL-1*β* increased by 3.3-fold and pro-IL-18 by 3.8-fold at 4 hours post adenosine induction when compared to control cells (Figure 3(d)). However, intracellular pro-IL-1*β* levels were undetectable by immunoblotting. Although QPCR data of IL-1*β* and IL-18 only suggest that NF-*κ*B is activated, we did not see any change in Rel A, a subunit of NF-*κ*B (Figure 3(e)). Taken together, the absence of extracellular IL-1*β* and IL-18 in addition to the decrease of intracellular caspase-1 and IL-1*β* after adenosine treatment suggests that adenosine suppresses the conversion of pro-caspase-1 and pro-IL-1*β* to their mature forms in Daudi cells.

### 3.4. Coculturing with NRLP11 siRNA-Transfected Daudi Cells Restored Th1 and Th17 Cell Responses but Did Not Alter Th2 and Treg Responses

**Figure 4:**
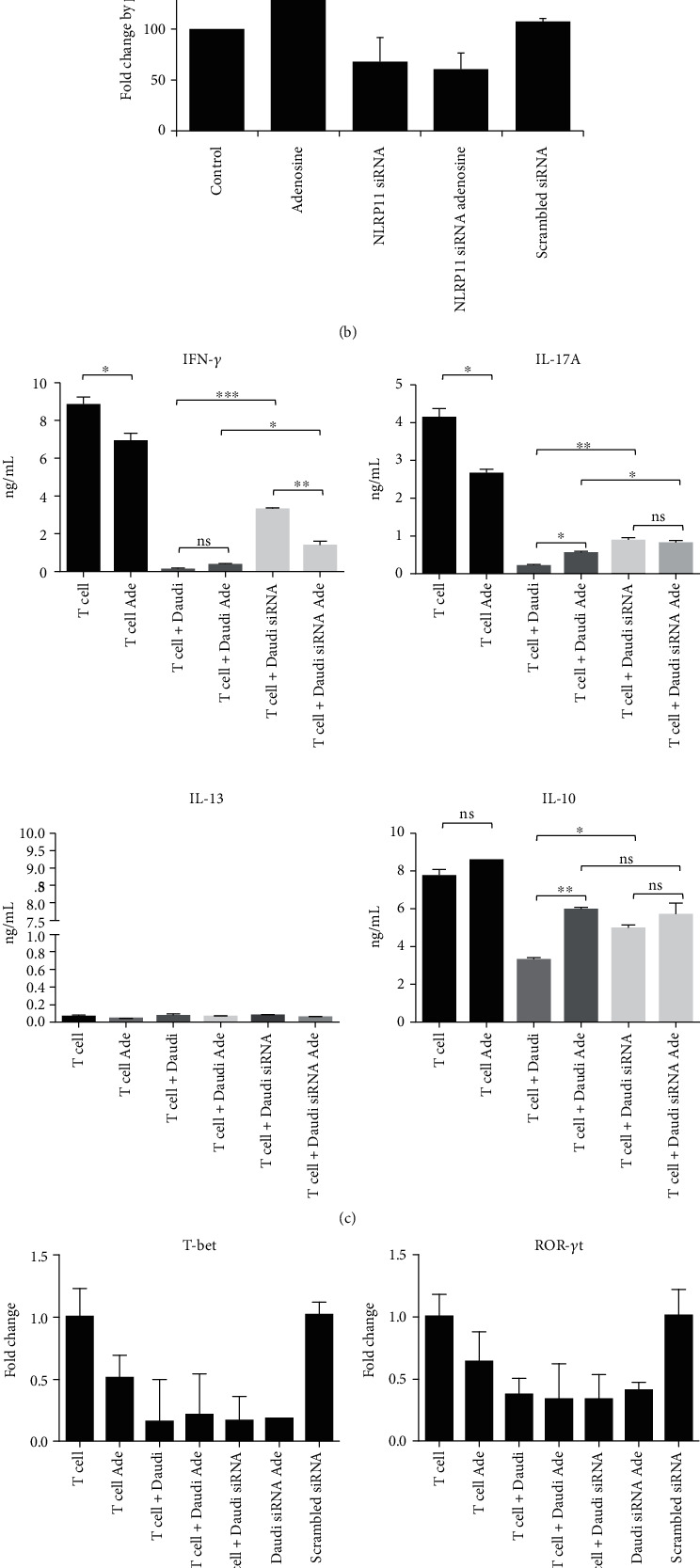
Coculturing with NRLP11 siRNA-transfected Daudi cells restored Th1 and Th17 cell responses but did not alter Th2 and Treg responses. (a) Costimulatory molecules CD80 and CD86 surface expressions were determined by a flow cytometer. (b) NLRP11 mRNA expression in Daudi cells that were transfected with scrambled siRNA or cotransfected with the mixture of S1, S2, and S3 siRNAs. Data are presented as percentages compared with the negative siRNA. (c) 24 hours after transfection, Daudi cells were treated with adenosine and cell and supernatants were harvested following 4 hours of stimulation. IFN-*γ*, IL-17A, IL-10, and IL-13 levels were measured in the supernatants by ELISA. (d) T-bet, GATA3, ROR*γ*t, and FOXP3 gene expressions were measured by QPCR. Data are presented as fold changes compared with the control. Experiments were carried out in duplicates. Results are representative of three independent experiments. Fold changes were depicted on the *Y* axis, which shows the significant difference between negative siRNA transfected and siRNA transfected. Values represent the mean ± SD and are representative of four separate experiments. Student's *t*-test shows the significant difference between stimulated and nonstimulated cells. ^∗^ indicates *P* < 0.05, ^∗∗^*P* < 0.01, and ^∗∗∗^*P* < 0.001.

Inhibition of T-cell functions by B cells is dependent upon the activation state and surroundings of the latter [33]. Moreover, B cell-derived adenosine or components of the adenosine pathway are of great importance in regulating T cell functions, as all B cells exhibit this particular regulatory function, and not just small B cell-like subsets [34]. Because Daudi cells bear CD80 and CD86 as costimulatory molecules on their surface, we utilized these cells as APCs in blood-derived human primary CD4^+^ T cell cocultures. We then investigated the extent to which NLRP11 promoted the differentiation of CD4^+^ T cells into effector T cells (Figure 4). To evaluate the roles of NLRP11 in shaping adaptive immune responses with an emphasis on the T helper cell polarization, we utilized cocultures of Daudi cells and naïve primary human CD4^+^ T cells, the latter derived from peripheral blood of healthy donors. In this particular experiment, Daudi cells served as Antigen Presenting Cells (APCs) due to their ability to express costimulatory molecules such as CD80 and CD86. Moreover, siRNAs against NLRP11 did not have any influence on the costimulatory molecule expression (Figure 4(a)). While costimulatory molecules are expressed on the surface of Daudi cells, they do not express CTLA-4 and PD-L1 inhibitory molecules [32].

Most importantly, Daudi cells have been shown to have a role in *γδ* T-cell expansion via an interaction with human leukocyte antigen (HLA) class II on their surface [35]. Collectively, all these data, including the relatively high expression of NLRP11, made Daudi cells an ideal coculture partner for human peripheral primary CD4^+^ T cells. To determine the NLRP11 dependency of Th1, Th2, Th17, and Treg cellular immune responses, we utilized short interfering RNAs (siRNAs) directed at NLRP11 and transfected Daudi cells with these siRNAs. Fold changes depicted as percentages and transfection of cells with siRNAs targeting NLRP11 resulted in a 40% reduction in NLRP11 mRNA expression (Figure 4(b)). The transfected Daudi cells were then transferred onto human primary T cell cultures. T helper cells are typified by their elaboration of proinflammatory cytokines IFN-*γ* for Th1; IL-13, IL-5, and IL-4 for Th2; IL-17A for Th17; and IL-10 and TGF-*β* for Treg cells. Therefore, we measured the production of IFN-*γ*, IL-13, IL-17A, and IL-10 levels in these cocultures (Figure 4(c)). Human peripheral CD4^+^ T helper cells produced significantly less IFN-*γ* and IL-17A when cocultured with NLRP11-expressing Daudi cells, and interestingly, these responses were significantly restored when CD4^+^ T helper cells were cocultured with NLRP11 siRNA-transfected Daudi cells (*P* < 0.05), suggesting a role for NLRP11 in suppressing Th1 and Th17 cell responses. While we did not observe any IL-13 production in any of the treatment groups, we were able to measure IL-10 production in cocultures of NLRP11-expressing Daudi cells and naïve blood-derived primary human CD4^+^ T cells; however, these IL-10 responses were not restored in the cocultures of NLRP11 siRNA-transfected Daudi cells and naïve blood-derived primary human CD4^+^ T cells (Figure 4(c)). Collectively, these findings suggest an extrinsic effect of NLRP11 in the regulation of T helper cell responses. We next evaluated the expression of specific transcription factors (TFs) including T-bet, GATA3, ROR*γ*t, and FOXP3 as hallmarks of Th1, Th2, Th17, and Treg cells, respectively (Figure 4(d)). Of all the TFs tested, T-bet and RORC mRNA expression were significantly downregulated in NLRP11-expressing cocultures and showed a similar trend to IFN-*γ* and IL-17A cytokine production. Although we did not see a significant recapitulation in the mRNA expressions of T-bet and RORC, these data support the suppression of Th1 and Th17 in NLRP11-expressing cocultures.

### 3.5. Adenosine-Induced NLRP11 Might Be Operating through the A_2B_ Receptor Signaling Pathway

**Figure 5:**
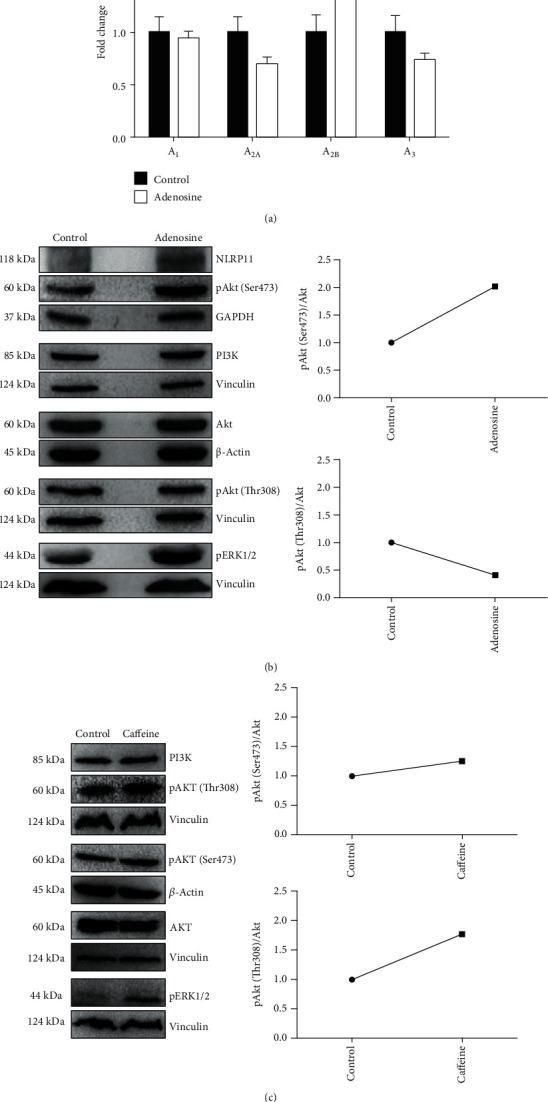
Adenosine induces NLRP11 and A_2B_ receptor gene expression and elevates endogenous ERK1/2 and phosphorylated Akt protein levels in Burkitt's lymphoma cells. (a) A_1_, A_2A_, A_2B_, and A_3_AR fold changes in adenosine-induced human Daudi cells at 4 hours post stimulation. (b and c) After adenosine and caffeine treatment, cell lysates were harvested and total protein was separated by SDS-PAGE. Blots were probed with anti-NLRP11, ERK1/2, Akt, pAkt (Thr308), and pAkt (Ser473) antibodies and visualized using ECL after incubation with an anti-rat HRP-conjugated antibody. Images are representative of two independent experiments.

Adenosine receptor signaling pathways are involved in various cellular responses such as proinflammatory or anti-inflammatory responses, cell survival, and tissue repair [9, 12]. ERK1/2 and Akt signaling pathways are the most studied and characterized molecular mechanisms upon adenosine receptor activation and can have numerous roles in mediating the proinflammatory and anti-inflammatory responses. Adenosine initiates its biological effects via four receptor subtypes, namely, A_1_, A_2A_, A_2B_, and A_3_ARs. Here, we measured mRNA levels of these genes in Daudi cells after adenosine stimulation and its subsequent influence on the ERK1/and Akt signaling pathways (Figure 5). Daudi cells are known to express the A_2A_ receptor more abundantly than the other subtypes of adenosine receptors at the steady state level [32], which was consistent with our findings as well. However, of all the adenosine receptors we measured, A_2B_ was the only receptor that was induced after 4 hours post adenosine stimulation as compared to the control (Figure 5(a)).

**Figure 6:**
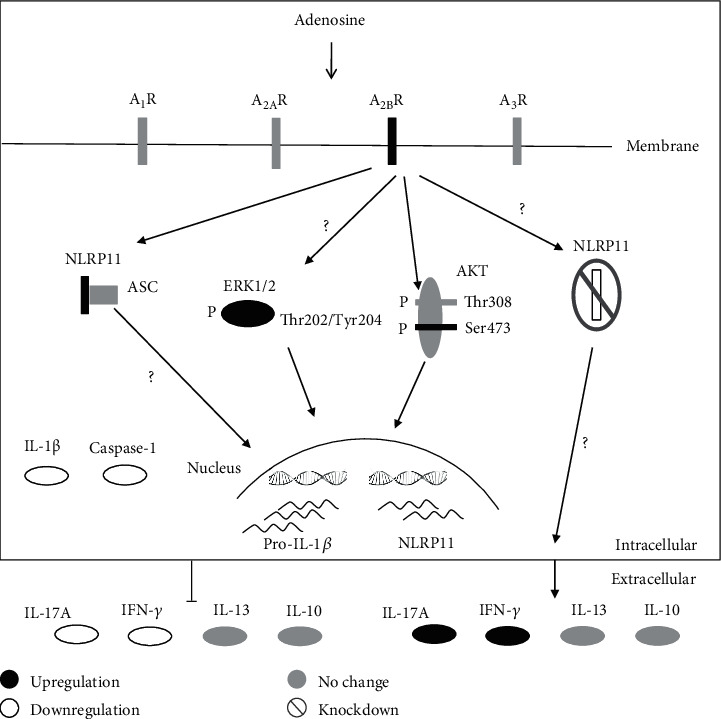
Schematic diagram of the regulation of adenosine response via NLRP11 in humans.

Adenosine receptors are G protein-coupled receptors whose induction entails adenosine stimulation and results in the accumulation of cyclic adenosine monophosphate (cAMP). cAMP has been suggested to have an important role in the inflammatory responses by triggering different downstream signaling pathways including PI3K/AKT (phosphoinositide 3-kinase/protein kinase B) and mitogen-activated protein kinase (ERK1/2) [36]. Once adenosine binds its receptor, it initiates a cascade, leading to changes in the phosphorylation of AKT and ERK1/2 via separate pathways. Furthermore, the adenosine A_2A_ receptor has been shown to regulate Akt and ERK1/2 pathways in a tissue- and cell-specific manner [37]. Phosphorylation is crucial for maintaining signaling homeostasis. Phosphorylation or dephosphorylation of a protein is a reversible mechanism that can alter its conformation and functional stability [38]. It can also affect the rate of protein degradation and translocation within the cell from one compartment to another [39]. Therefore, we reasoned that adenosine treatment might alter the phosphorylation states of ERK1/2 and Akt pathway components along with NLRP11 induction (Figure 5). Phosphoproteomics data showed that threonine phosphorylation and tyrosine phosphorylation are remarkably less (approximately 15% and 2%, respectively) than the phosphorylation of serine residues (approximately 85%) [40, 41]. Although the phosphorylation of Akt at its catalytic site Thr308 activates the kinase, the phosphorylation of the hydrophobic residue Ser473 further increases the enzymatic activity of the enzyme and expands its substrate spectrum [42, 43]. We sought to investigate the changes in Akt and ERK1/2 phosphorylation to identify the downstream effectors of the adenosine signaling pathway that leads to the induction of NLRP11 (Figure 5(b)). We found that adenosine treatment significantly upregulated phosphorylated Akt and ERK1/2 levels as compared to controls, and exerted a positive impact on NLRP11 expression. Akt is deemed to require phosphorylation at Ser473, along with Thr308 within the carboxy terminus; however, the regulatory mechanisms and importance of each phosphorylation site need to be further dissected. Most of many studies have shown that Akt is simultaneously phosphorylated at multiple sites [44–46]. Interestingly, here we observed that levels of Akt phosphorylated at Ser473 were increased, but not Thr308 upon adenosine treatment in Daudi cells (Figure 5(b)). Additionally, caffeine treatment did not alter the levels of Akt that are phosphorylated at Ser473; however, the phosphorylated Thr308 version of Akt increased upon caffeine treatment (Figure 5(c)), representing a unique pathway associated with the induction of NLRP11 expression upon adenosine treatment (Figure 6). Interestingly, the roles of anti-inflammatory cytokine IL-10 were previously shown for the progression of B cell lymphoma [47]. These findings suggest a positive correlation between adenosine A_2B_ receptor occupancy on the one hand, and Akt and ERK1/2 phosphorylation and NLRP11 expression on the other.

## 4. Conclusion

It is noteworthy that even though adenosine and NLRP11 are both reported to have anti-inflammatory roles, the positive stimulatory effect of adenosine on NLRP11 in B lymphoblast cells might reveal a new role for NLRP11 in cancer development and progression. Overall, our findings raise the possibility of new targets for the treatment of immune system-related diseases originating from deficient lymphocyte responses and lay the groundwork for further studies to understand the mechanism by which NLRP11 regulates T cell polarization.

## Data Availability

The data used to support the findings of this study are included within the article.
